# Exploring the Acute Effects of Stimulating Different Muscle Combinations During Neuromuscular Electrical Stimulation-Assisted Treadmill Walking in Children with Cerebral Palsy

**DOI:** 10.3390/bioengineering13101193

**Published:** 2026-10-14

**Authors:** Mobina Masaei, Nicole Zahradka, Khushboo Verma, Samuel C. K. Lee, Ahad Behboodi

**Affiliations:** 1 Department of Biomechanics, University of Nebraska Omaha, Omaha, NE 68182, USA; mmasaeikoroyeh@unomaha.edu; 2 Pediatric Mobility Laboratory, Department of Physical Therapy, University of Delaware, Newark, DE 19716, USA; nicole.zahradka@gmail.com (N.Z.); vkhush@udel.edu (K.V.); slee@udel.edu (S.C.K.L.)

**Keywords:** CP, NMES, gait analysis, Gait Deviation Index (GDI), Coefficient of Variation (CV), Dynamic Time Warping (DTW)

## Abstract

Progressive gait impairments remain a major challenge for children with cerebral palsy (CP), often limiting independence and quality of life. Although neuromuscular electrical stimulation (NMES) has shown promise for improving gait, most NMES-assisted gait studies in children with CP have targeted only a single muscle. In this exploratory study, we characterized acute biomechanical responses of six children with CP to seven NMES protocols during treadmill walking. The protocols comprised multi-muscle stimulation, single-muscle stimulation, and an individualized physical therapist-derived (PT-derived) condition. Each protocol was compared with a no-stimulation baseline and typically developing (TD) reference data. The primary and secondary outcomes were the Gait Deviation Index (GDI) and Coefficient of Variation (CV), complemented by the targeted outcome measures, including lower-limb joint kinematics using phase-by-phase Dynamic Time Warping (DTW), maximum knee extension, and stride length. We found that the comparison between multi-muscle and single-muscle conditions did not identify a statistically significant difference in GDI. CV analyses showed smaller distances from TD reference values for multi-muscle conditions, although not significant. Stimulation conditions significantly affected maximum knee extension and DTW during loading response at the ankle and pre-swing at the hip; however, no individual condition differed significantly from no stimulation after false discovery rate correction. The PT-derived condition showed favorable directional changes in selected outcomes, but no consistent advantage over the other protocols. These preliminary observations characterize acute response patterns and generate hypotheses for future studies; they do not establish the comparative efficacy of multi-muscle, single-muscle, or individualized NMES protocols.

## 1. Introduction

Cerebral Palsy (CP) is the most prevalent childhood neuromotor diagnosis, with between 550,000 and 764,000 current cases in the US [1]. The natural progression of CP is characterized by a gradual decline in walking function, which negatively impacts all three components of the WHO ICF model of disability: body function and structure, activity and participation, and ultimately, quality of life. Walking function in individuals with CP declines through adolescence and into adulthood [2,3]. Children with CP have improper muscle timing and duration compared to the muscle activity of typically developing (TD) children [4] and diminished extension during walking (also known as crouch). This results in a shorter stride length and a slower walking speed [5] and is associated with expending two to three times more metabolic energy while walking than their TD peers [6]. Existing interventions address different contributors to gait impairment in CP, and their effects on walking outcomes vary. This variability motivates the investigation of complementary approaches that assist muscle activation during walking [7,8,9,10]. Thus, interventions that improve lower-limb muscle contractions to maintain upright posture during gait are crucial in CP.

Neuromuscular electrical stimulation (NMES) is a physiologically based intervention that augments muscle contractions during active movement by applying electrical stimulation through the skin. By providing appropriately timed and powered muscle contractions, NMES can help compensate for deficits in selective motor control in individuals with neurological disorders, leading to more efficient gait mechanics [11]. Additionally, NMES activates Golgi tendon organs and muscle spindles, offering rich sensory feedback via afferent neural pathways. This stimulation engages multiple motor control components, impacts cortical excitability, and may promote motor learning [12]. These characteristics make NMES particularly relevant to gait rehabilitation because it can assist the participant’s own muscle activation during walking.

A recent meta-analysis demonstrated a large effect size in improving gross motor function when NMES was used in the motor rehabilitation of children with CP [13]. Emerging NMES efforts for children with CP suggest improvements in spatiotemporal parameters [14,15,16,17], kinematics [14,18,19,20], and kinetics [18] during walking. However, much of the NMES literature involving children with CP has focused on individual muscles or joints, despite the multi-joint nature of their gait deviations [12]. Studies reported that kinematic improvements occurred based on the muscle groups targeted: stimulation to the triceps surae (TS) [21], dorsiflexors [14,18,19,20,22,23], or gastrocnemius and tibialis anterior (TA) [21,24] promoted changes at the ankle; stimulation to the quadriceps [18,25] improved knee flexion angle, and continuous low-level stimulation of the gluteus medius [26] improved hip abduction. These findings demonstrate that the biomechanical response to NMES depends on the muscles targeted. At the same time, assisting a single muscle may be insufficient to address deviations that involve coordinated movement across the hip, knee, and ankle. Coordinated stimulation of multiple muscles may produce broader biomechanical responses than isolated muscle stimulation. For example, combined stimulation of the rectus femoris and vastus lateralis produced greater improvement in posture than stimulation of either muscle alone during walking in one child with CP [27].

Previously, to investigate the effect of multi-muscle stimulation, our group developed a multi-muscle system capable of stimulating up to five muscle groups on each leg during gait [28,29,30,31]. We evaluated the acute (neuroprosthetic) effects [30] and therapeutic (neurotherapeutic) effects [32] of this NMES system. In a 12-week gait training protocol on two children with CP, we demonstrated alterations toward typical gait in hip and ankle angles, which lasted without NMES for four weeks post-training [32,33]. Rose et al. employed a multichannel approach and reported similar acute results in three children with CP [34]. However, evidence of multi-muscle NMES effectiveness is still limited [12].

As multi-muscle NMES-assisted walking interventions for children with CP are still in their early stages of development, identifying optimal muscle combinations for stimulation is essential to designing an effective intervention. Previous studies have typically applied a fixed stimulation protocol across participants. This standardization, given the heterogeneity of gait deviations in the CP population, may reduce potential effectiveness and, in turn, our comprehensive understanding of the intervention. The contribution of this study is twofold: (1) it provides a systematic comparison of multiple multi-muscle and single-muscle NMES protocols, and (2) it contrasts an individualized, physical therapist (PT)-derived protocol with uniform approaches. The PT-derived protocol is a subject-specific, multi-muscle protocol designed by PTs to target all three lower limb joints based on the individual’s gait deviations, representing a key distinguishing feature of this work. All stimulation protocols (NMES conditions) were compared to walking without stimulation and the gait pattern of TD individuals to characterize condition-specific differences in similarity to typical gait kinematics.

This exploratory study examined two research expectations: (1) coordinated multi-muscle stimulation would be associated with more favorable acute biomechanical responses than single-muscle stimulation and (2) an individualized PT-derived multi-muscle NMES protocol would be associated with more favorable responses than standardized multi-muscle protocols. Overall, the objective was to characterize the magnitude and direction of within-participant responses and identify patterns warranting further investigation, rather than establish comparative efficacy. This paper presents the study design, including details of the stimulation protocols, the NMES-assisted walking system, and the outcome measures employed. The Gait Deviation Index (GDI) was designated as the primary outcome because it provides a summary measure of overall gait pathology, and the coefficient of variation (CV) was the secondary outcome measure. Phase-specific Dynamic Time Warping (DTW), maximum knee extension, and stride length were included as complementary measures to characterize joint- and phase-specific responses.

## 2. Materials and Methods

### 2.1. Participants: Intervention Group

Children were eligible to participate in this study if they were diagnosed with spastic diplegic CP, aged between 7 and 18 years old, had Gross Motor Function Classification System (GMFCS) levels II or III, and showed at least 0° of passive ankle dorsiflexion. Exclusion criteria included severe leg-muscle spasticity, defined as a Modified Ashworth Scale score of 4; knee flexion contracture greater than 10° or hip flexion contracture greater than 15°; lower-extremity surgery or fracture within the preceding year; and botulinum toxin injections into lower-extremity muscles within the preceding six months. After advertising Institutional Review Board (IRB)-approved recruitment flyers through outpatient CP clinics and local referral channels, we recruited six children with the characteristics detailed in Table 1. Prior to participation, parental consent and child assent were obtained from all volunteer participants. Subsequently, their height and weight were measured.

### 2.2. Participants: Control Group

The TD control group comprised seven children recruited locally, including five females and two males, with a mean age of 12.43 ± 2.15 years. Mean height was 1.58 ± 0.13 m, and mean body mass was 46.43 ± 14.71 kg. Parental consent and child assent were obtained before participation under the approved study procedures. Participant characteristics are provided in Table 1. These children provided a study-specific reference dataset for contextualizing gait characteristics in the CP participants.

### 2.3. Study Design

**Figure 1:**
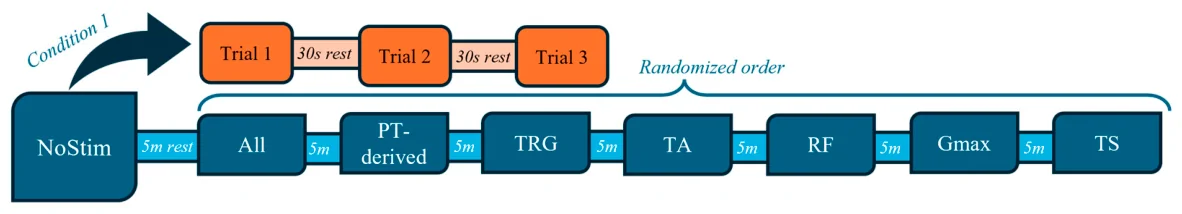
Block diagram of study design. The study included eight conditions; seven conditions included stimulation applied to different muscle combinations: All = typical muscle activation patterns; PT-derived = physical therapists’ chosen muscles; TRG = combined activation of Triceps Surae, Rectus Femoris and Gluteus Maximus (i.e., prominent lower limb extensor muscle groups); TA = Tibialis Anterior; RF = Rectus Femoris; Gmax = Gluteus Maximus; and TS = Triceps Surae. The session started with the NoStim (without any stimulation) condition, while the order of stimulation conditions was randomized between participants, with each condition repeated three times.

To explore the effectiveness of different muscle combinations in improving gait kinematics, this study comprised a single session in which each participant with CP completed seven distinct stimulation protocols while walking on a treadmill, hereinafter referred to as NMES conditions, plus a no-stimulation condition as the baseline. Each condition lasted approximately 1 min and was repeated three times (a total of 24 trials), with a 5-min rest interval between conditions and a 30-s rest between trials (Figure 1). Participants who were unable to complete all trials of the conditions in a single session returned the following day. The NMES conditions were as follows:

1. Baseline: Participants walked on the treadmill without NMES. As there was no electrical stimulation, we called this condition “NoStim”.
2. Literature-derived: Participants walked on the treadmill while receiving NMES assistance based on typical muscle activation patterns during walking. These patterns were derived from typical lower limb Electromyography (EMG) data during gait reported in the existing literature [35]. The targeted muscle groups included TA, RF, Gluteus Maximus (Gmax), and Hamstring (Ham). All participants received NMES using a uniform protocol, which we referred to as the “All” condition.
3. PT-derived: Three physical therapists experienced in identifying gait deviations in children with CP reviewed frontal and sagittal walking videos containing at least ten consecutive steps. The therapists subsequently compared their separate observations and resolved disagreements through discussion. Consensus required agreement on the gait deviations to address, the muscle groups to stimulate, and the timing and level of stimulation. Muscle selection and gait-phase timing were guided by clinical judgment [30]. Stimulation settings were established through the functional thresholding procedure and could be adjusted during walking according to the observed response and participant tolerance. Supplementary Table S1 presents the participant-specific treatment targets, muscle-phase selections, prescription rationale, and relevant modifications.
4. TRG: Participants walked on the treadmill while receiving NMES assistance to the prominent lower-limb extensor muscles: TS, RF, and Gmax.
5. TA: Participants walked on the treadmill, receiving NMES assistance solely for the TA muscle.
6. RF: Participants walked on the treadmill while NMES was solely applied to the RF muscle.
7. Gmax: Participants walked on the treadmill while NMES was solely applied to the Gmax muscle.
8. TS: Participants walked on the treadmill while NMES was solely applied to the TS muscle.

The stimulation conditions were classified according to the number of targeted muscle groups. The All, PT-derived, and TRG conditions targeted more than one muscle group and were classified as multi-muscle NMES. The TA, RF, Gmax, and TS conditions each targeted one muscle group and were classified as single-muscle NMES. NoStim served as the unstimulated baseline and was not included in either stimulation category. All participants completed the NoStim baseline before the seven stimulation conditions, whose order was randomized, in MS Excel for each participant (Figure 1). Participants were unaware of the order of conditions beforehand. However, due to the significant sensory component of the NMES, they could feel which muscle was being stimulated during the trials. Placing NoStim first provided a baseline before exposure to the experimental stimulation conditions and reduced the possibility of carryover from those conditions into the baseline measurement. This approach did not counterbalance baseline and stimulation conditions and therefore could not eliminate order-related influences on comparisons with NoStim. During all conditions, subjects walked at their self-selected walking speed (SSWS) on the treadmill with handrails on each side for participants to use. Orthoses and habitual assistive devices were not used during experimental treadmill walking.

TD participants completed three 30-s walking trials without stimulation on the instrumented treadmill at their SSWS. The same laboratory acquisition setup used for the CP participants was employed [30]. An eight-camera motion capture system (Motion Analysis Corporation, Santa Rosa, CA, USA) with a sampling rate of 128 Hz was utilized to capture gait kinematics. Kinetics were obtained using two force plates (Bertec, Columbus, OH, USA) with a sampling rate of 3200 Hz. To ensure full-body movement capture, 33 reflective markers were placed on anatomical landmarks, including the shoulders, trunk, pelvis, legs, and feet.

To identify the more affected side, each subject’s ankle, knee, and hip joints were assessed, with the side showing the lowest active and passive range of motion designated as the more affected side. The SSWS was initially derived from the 10-m walk test (10MWT) overground. A comfortable treadmill speed was then established based on participant feedback; if the overground speed felt too fast on the treadmill, it was adjusted to a more comfortable pace. A non-weight-bearing harness was utilized to mitigate the risk of stumbles or falls.

### 2.4. System

#### 2.4.1. Neuromuscular Electrical Stimulation

In this study, the core technology we employed was our previously customized multi-muscle NMES-assisted gait training system with the capability of targeting up to 10 muscle groups bilaterally during each phase of gait: left and right TA, RF, Gmax, Ham, and TS [28,29,30,31]. The system setup consisted of two inertial measurement units (IMUs) (OpalTM, APDM, Portland, OR, USA) placed on the shank, two stimulators (RehaStim, Hasomed Inc., Magdeburg, Germany), and a custom gait phase detection (GPD) algorithm [29] implemented in LabVIEW (National Instruments, Austin, TX, USA). The GPD algorithm identified gait phases by analyzing wireless inputs of left and right shank angular velocities, enabling finite-state control of stimulation timing. Using predefined rules derived from angular velocity profile features, the algorithm distinguished the seven phases of gait: loading response (LR), mid-stance (MSt), terminal stance (TSt), pre-swing (PS), initial swing (ISw), mid-swing (MSw), and terminal swing (TSw) [29]. Validation in children with CP demonstrated accurate performance, with root mean square errors for phase onset detection ranging from 63 ms (LR and PS) to 127 ms (MSw) when compared with motion capture.

By incorporating the GPD system, designed specifically for the CP population, this system could independently modulate the stimulation intensity over each gait phase for each muscle, resulting in increased customization flexibility. The LabVIEW-based GPD system utilized an application programming interface (API) to enable real-time updates to the stimulation parameters. This included adjustments to stimulation delivery timing, stimulation intensity (current and pulse duration), and the selection of target muscles for stimulation (stimulation channels), all while subjects walked on a treadmill. The system delivered charge-balanced biphasic stimulation at a fixed frequency of 40 Hz. Each muscle group was stimulated only during the gait phases specified by its protocol, rather than continuously throughout the walking condition. A pair of 2 × 2-inch electrode pads was used for the TA, and a pair of 3 × 5-inch pads was used for each of the TS, RF, and Gmax muscle groups.

To compensate for processing and hardware communication delays, the controller used the onset of the preceding gait phase to schedule stimulation near the onset of the target phase. The gait-phase detection algorithm identified phase onsets in real time and updated its phase-duration estimates as the participant walked. After detecting the onset of the preceding phase, the controller issued the stimulation command when 75% of that phase’s estimated duration had elapsed. Thus, the trigger was based on an updated duration estimate rather than a fixed delay in milliseconds. We previously evaluated this threshold against gait-phase onsets identified by motion capture and found that, on average, it placed stimulation closest to the intended phase onset among the thresholds tested [28].

#### 2.4.2. Adjusting Stimulation Intensity

We precisely determined the subject-specific functional stimulation intensity for each of the five bilateral lower limb muscle groups, resulting in a total of 10 intensities. This process, known as thresholding, involved two key steps for each muscle: (1) determining the motor threshold (MT) and (2) identifying the functional intensity level. The setup for stimulation delivery involved placing electrodes according to the Robinson and Snyder-Mackler protocol [36] on muscles targeted by each stimulation program. To ensure accurate electrode placement and stimulation thresholding, a preliminary low-level stimulation was administered.

To determine the MT of a muscle, the subject stood or sat to relax the target muscle. The stimulation current was initially set at 30 mA. The pulse duration was then gradually increased from zero in 5 µs increments until a strong muscle twitch, indicating MT, was observed. If no muscle twitch occurred by the time the pulse duration reached 250 µs, the current was increased by 10 mA, and the pulse duration was again incrementally increased from zero. The stimulation frequency was set at 40 Hz. To identify the functional stimulation level of the muscle, the stimulation train duration for each phase was set to match the duration of the target gait phase (typically 50–300 ms), measured during the “NoStim” condition. The subject then assumed the position corresponding to the target phase, and the pulse width and current were increased incrementally from the MT intensity, in a manner similar to MT identification, until either the functional goals specific to the phase and muscle were achieved or the subject’s maximum tolerance was reached.

For instance, to adjust the functional level of stimulation for the TS muscles, the participants stood and positioned their lower limbs as they would during the PS phase. The stimulation intensity was increased above the MT until the muscle contraction could lift the heel off the ground, representing the TS’s phase-specific functional role. The detailed thresholding procedure is published as Supplementary Material [30].

Thus, for every stimulation condition, current was set separately for each participant, muscle group, and limb. Pulse duration could also vary by gait phase. Starting from the motor-threshold settings, we increased stimulation until the intended movement was achieved or the participant reached their maximum tolerated intensity. We did not prescribe intensity as a fixed percentage of motor threshold. When a lower stimulation level was selected, pulse duration was set to approximately 50% of the pulse duration that produced the functional response. Participant-specific current and pulse-duration ranges are summarized in Table 2.

In the individualized PT-derived programs, active current settings ranged from 30 to 90 mA, and pulse durations ranged from 102 to 470 µs. The detailed information on individual stimulation protocols can be found in Figure S1 of the Supplementary Material.

If stimulation thresholding occurred the day prior to gait analysis, markings were made on the legs to indicate the corners of each electrode. These markings served as reference points for electrode placement the following day, when stimulation intensities were reassessed.

### 2.5. Outcome Measures

The primary goal of this study was to evaluate the overall influence of NMES on gait performance, rather than to correct isolated moments within the gait cycle. Although NMES was delivered at specific gait phases, our intent was to determine whether these localized interventions translated into global improvements in walking patterns. Accordingly, our primary outcome measure, the GDI, was selected for its ability to capture overall gait quality. A limited set of targeted outcome measures was also included, not to shift the focus toward single-muscle corrections but to examine whether improvements at targeted joints or phases meaningfully translate into broader, global gait improvements. Notably, based on our prior work [30], the effects of NMES on targeted outcomes vary considerably across individuals, which can dilute their influence on whole-gait performance; thus, GDI provides a more stable metric for comparing NMES conditions.

#### 2.5.1. Global Summary Outcome Measures

##### Gait Deviation Index

The primary outcome measure in this study was the GDI of the more affected side (left for subjects 1, 2, 5, and 6 and right for subjects 3 and 4). GDI is a reliable and comprehensive index of gait pathology based on 15 different kinematic measures (bilaterally) or nine unilaterally, including:Knee Flexion/ExtensionAnkle Dorsiflexion/PlantarflexionPelvic TiltPelvic RotationPelvic ObliquityHip RotationHip Abduction/AdductionHip Flexion/ExtensionFoot Progression

The similarity of our participants’ gait (CP; the intervention group) to that of the control group, TD individuals in this study, was measured by the scaled distance of the kinematic measures from those of TDs [37]. This index has been proven to be effective in capturing overall gait kinematic changes in children with CP [34,38].

GDI of 100 represents the TDs’ GDI score. An increase of ten GDI units corresponds to one standard deviation (SD) increase in gait similarity between CP subjects and those of controls [37]. An increase of 5.0 points is considered a clinically meaningful difference (CMD) in GDI [34]. The direction of kinematic improvements was defined as getting closer to those measured for TDs, a GDI of 100.

##### Coefficient of Variation

The coefficient of variation (CV) shows how much a set of numbers varies relative to their average, without being affected by the unit of measurement. By dividing the SD by the mean, the CV allows for the comparison of variability across different datasets, even if they use different units. A higher CV indicates greater dispersion around the mean. In this study, we used CV as a secondary outcome measure to evaluate the effect of NMES on the lower limb joint angle variability in the sagittal plane. However, the conventional statistical CV is unsuitable for analyzing cyclical biomechanical data, such as joint angles, where the arithmetic mean over a full gait cycle can be at or near zero. This renders the standard CV calculation undefined or artificially inflated. Therefore, we employed the adapted CV proposed by D.A. Winter [39] which is a standard method in gait analysis. This ‘variability-to-signal ratio’ resolves this issue by replacing the problematic denominator with the mean of the absolute values of the signal, representing the average signal magnitude. This provides a stable, non-zero normalization factor, while the numerator represents the root mean square of the ensemble SD across the cycle [40].

Sagittal-plane ankle, knee, and hip angle waveforms were processed in Visual3D (C-Motion, Inc., Germantown, MD, USA). Each gait cycle, defined from initial contact to the subsequent ipsilateral initial contact, was time-normalized to 100% and resampled at 51 equally spaced points. For each participant with CP, Visual3D was then used to compute the ensemble mean and standard deviation across that participant’s gait cycles, separately for each joint and stimulation condition. At each normalized point, the mean represented the participant’s average joint angle, and the standard deviation represented cycle-to-cycle variability. We implemented the same procedure for the TD reference group, with the exception of not having different stimulation conditions. We individually inserted the values for each joint under each condition into the following equation to derive the CV values for individual joints:

(1) $$\mathrm{CV}=\frac{\sqrt{\frac{1}{N}\sum_{i=1}^{N}\sigma^{2}}}{\frac{1}{N}\sum_{i=1}^{N}\left|X_{i}\right|}$$

where *N* is the number of intervals over the stride, *X_i_* is the mean value of the variable at the *i^th^* interval, and *σ* is the standard deviation of variable *X* about *X_i_*. We calculated the CV for each TD subject; we measured the mean and SD of the resulting values to end up with one reference number.

To improve interpretability, GDI scores and the CV for each joint across all conditions were averaged for each participant, resulting in a composite metric denoted as $\bar{\mathrm{GDI}}$ and $\bar{\mathrm{CV}}$ respectively. The mean and standard deviation of $\bar{\mathrm{CV}}$ from the TD cohort were used to represent normative values on the graphs.

To better emphasize the changes from the baseline, we also subtracted the GDI from the “NoStim” condition and CV results from the “TD” results.

#### 2.5.2. Targeted Outcome Measures

Since global metrics such as GDI and CV average performance across the entire gait cycle, they may mask phase-specific or joint-level adaptations induced by NMES. Therefore, while standardized global indices were used to ensure consistent comparisons across stimulation protocols, we complemented them with targeted biomechanical measures to better capture the mechanisms directly influenced by stimulation. These included time-series analyses of ankle, knee, and hip kinematics using phase-specific DTW, maximum knee extension, and stride length. Particular emphasis was placed on the knee joint due to its central biomechanical role in gait efficiency, limb clearance, and stance stability. Reduced knee extension has been directly associated with increased metabolic cost in adolescents with CP [41], and longitudinal evidence indicates that knee contractures often develop earlier than hip or ankle contractures [42,43], underscoring the importance of early knee-focused intervention. By combining global indices with joint-level and temporal analyses, we aimed to determine whether localized improvements, especially at the knee, translated into broader functional gains.

##### Phase-by-Phase Evaluation: Dynamic Time Warping and Time Series Analysis of the Ankle, Knee, and Hip Joints in the Sagittal Plane

To quantify joint-specific and phase-specific adaptations, we performed DTW analysis on sagittal-plane ankle, knee, and hip kinematics. DTW is a time-series technique that measures similarity between two waveforms by allowing non-linear temporal alignment. Unlike traditional point-by-point comparisons, DTW stretches or compresses segments of a waveform to achieve optimal alignment, minimizing cumulative distance between signals. This makes it particularly suitable for gait analysis, where variations in stride duration or phase timing may occur even when overall movement patterns are similar.

Heel-strike (HS) and toe-off (TO) events of the more affected limb were identified using vertical ground reaction force (vGRF) thresholds (initial contact defined as vGRF > 20 N; TO defined as vGRF < 20 N). Gait cycles were defined from HS to subsequent HS and segmented into four phases: Loading Response (LR: HS to contralateral TO), Single Support Stance (SSt: contralateral TO to contralateral HS), Pre-Swing (PS: contralateral HS to ipsilateral TO), and Single Support Swing (SSw: ipsilateral TO to subsequent ipsilateral HS). All cycles were time-normalized to 100% using a custom MATLAB script to ensure consistent phase boundaries across subjects and conditions.

Sagittal-plane joint-angle waveforms were extracted from Visual3D. For each participant and stimulation condition, the waveform was compared with a study-specific TD mean waveform. In the knee analysis, both waveforms contained 51 time-normalized samples. Each waveform was independently standardized to a mean of zero and a standard deviation of one using MATLAB’s normalize function. We then calculated the distance using MATLAB’s dtw function with its default Euclidean local distance and without specifying a warping-window constraint. The resulting DTW value was the cumulative distance along the optimal alignment path; it was not divided by path length. Lower values, therefore, indicated greater similarity of standardized waveform shape after temporal alignment, rather than closer agreement in absolute joint angles or gait-event timing.

##### Maximum Knee Extension and Stride Length

To investigate phase-specific effects, maximum knee extension and its variability across conditions, along with stride length, were also quantified. Maximum knee extension angles were computed in Visual3D as the mean of the lowest knee angles, time-normalized to 100% of the gait cycle, measured across all gait cycles for each subject under each condition. Stride length was determined by calculating the mean distance from HS to the subsequent HS of the more affected leg, considering the treadmill SSWS of each subject, across all gait cycles for every condition. We considered the mean maximum knee extension (°) and mean stride length (m) across all gait cycles.

Our focus on the knee joint stems from its crucial role in influencing crouch gait and facilitating stance-to-swing transitions in children with CP. As shown in this study and our previous work [30,32], NMES configurations can produce selective improvements at individual joints or phases without uniformly affecting global metrics, underscoring the value of integrating both global and targeted measures to more fully characterize the mechanisms and effectiveness of multi-muscle and individualized NMES interventions.

#### 2.5.3. Statistical Analysis

Statistical analysis was performed on all the targeted outcome measures using MATLAB R2025b (MathWorks, Inc., Natick, MA, USA). Given the small sample size and repeated-measures design, the non-parametric Friedman test was used to assess differences among stimulation conditions. Significant Friedman tests were followed by pairwise comparisons using two-sided exact Wilcoxon signed-rank tests, contrasting each active condition against the “NoStim” baseline. For these active-condition versus NoStim comparisons, the false discovery rate (FDR) was controlled using the Benjamini–Hochberg procedure within each outcome-specific family of comparisons. A *p*-value of <0.05 was considered statistically significant for the Friedman tests, and an FDR-adjusted *p*-value of <0.05 was considered statistically significant for these baseline comparisons. Kendall’s coefficient of concordance (*W*) was reported as the effect size for Friedman tests, and matched-pairs rank-biserial correlation (*r_rb_*) was reported for pairwise comparisons. For pairwise comparisons in the DTW outcome measure, the change was calculated as the active-condition DTW minus the NoStim DTW; therefore, negative values indicated greater similarity to the TD reference waveform under stimulation.

The “Gmax” condition was omitted for CP05 due to the muscle’s heightened sensitivity and the subject’s reluctance to use NMES there. Consequently, this condition was excluded from the omnibus statistical analysis of maximum knee extension, stride length, and multi-muscle vs. single-muscle. This allowed complete repeated-measures analysis of the remaining conditions while retaining all six participants. Gmax was also excluded from the single-muscle category average for every participant to maintain consistent protocol composition in the multi-muscle versus single-muscle comparisons.

To explore differences between multi-muscle and single-muscle conditions, we first calculated the mean ± SD of the multi-muscle (“All”, “PT-derived”, and “TRG”) and single-muscle (“TA”, “RF”, and “TS”) $\bar{\mathrm{GDI}}$ values. These participant-level category averages were summarized using mean ± SD and compared using two-sided exact Wilcoxon signed-rank tests, without normality-based test selection. Matched-pairs rank-biserial correlation was reported as the effect size. For the CV, we first calculated the absolute difference between each subject’s joint CV in each condition and the corresponding TD reference CV, effectively using the TD values as a baseline for our analysis. Next, for each subject, we averaged these absolute distances from the TD across conditions, dividing them into two groups: multi-muscle and single-muscle $\bar{\mathrm{CV}}$. We followed the same statistical procedures as used for the $\bar{\mathrm{GDI}}$ to evaluate which group’s $\bar{\mathrm{CV}}$ was significantly closer to the TD values in each individual joint. Smaller distances indicated greater similarity to TD CV values, rather than necessarily lower variability.

Additionally, to better understand the overall effect of multi-muscle versus single-muscle conditions, we calculated the composite Euclidean distance from the TD across all three joints for every condition and then averaged those Euclidean distances within the two respective groups. For each multi-muscle versus single-muscle comparison, the mean paired difference was calculated as multi-muscle minus single-muscle. Positive differences indicated higher GDI under multi-muscle stimulation, whereas negative differences indicated smaller CV distances from TD values. Approximate 95% percentile bootstrap confidence intervals for the mean paired differences were calculated using 50,000 resamples of participant-level paired differences, preserving the paired design. TD reference values were treated as fixed. These intervals estimate mean differences and are distinct from the rank-based Wilcoxon test; their exclusion of zero need not agree with the test’s significance. Their precision and coverage remain uncertain with six participants.

The category comparisons used unadjusted *p*-values and confidence intervals, with *p* < 0.05 used as an exploratory threshold. Sensitivity to individual participants was additionally described using the range of mean paired differences obtained after omitting each participant in turn.

All statistical comparisons were interpreted within an exploratory framework. Interpretation considered the magnitude and direction of observed differences, the reported effect-size estimates, and participant-specific responses alongside *p*-values. GDI remained the primary outcome, while the complementary outcomes were used to identify response patterns and generate hypotheses. Given the small sample and the number of conditions and outcomes examined, statistically significant findings were not interpreted as establishing the general superiority of a stimulation strategy. Nonsignificant findings were not interpreted as evidence of equivalence.

## 3. Results

### 3.1. GDI

**Figure 2:**
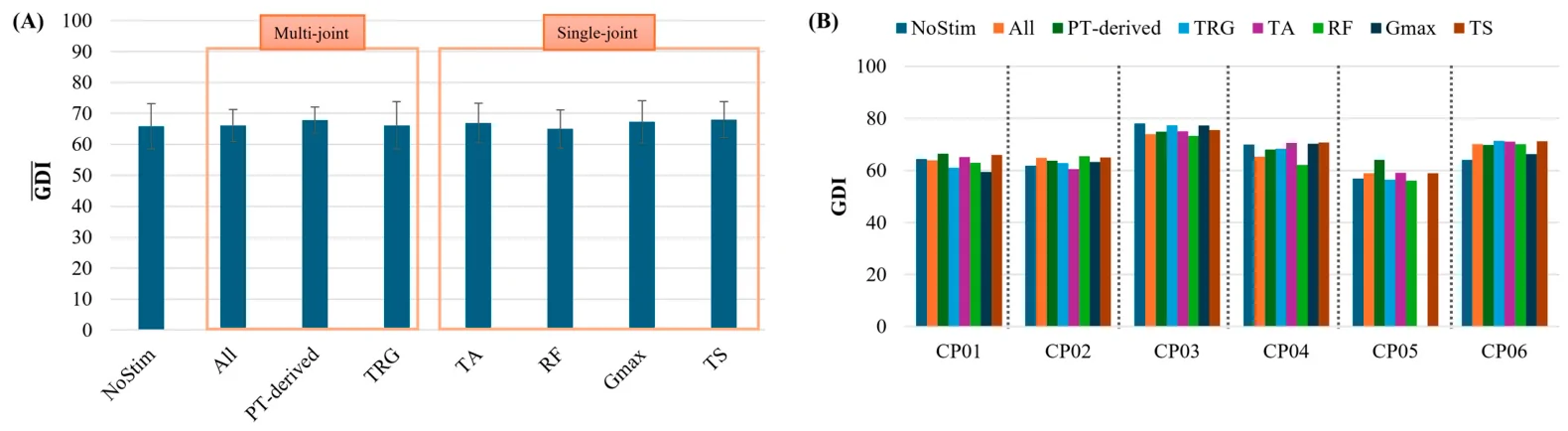
(**A**) $\bar{\mathrm{GDI}}$ represents the mean Gait Deviation Index (GDI) for each condition across all subjects, with highlighted multi-joint and single-joint NMES protocols. (**B**) Shows GDI values for each condition for each participant. Each color corresponds to a different condition. NoStim = without any stimulation; All = typical muscle activation patterns; PT-derived = physical therapists’ chosen muscles; TRG = combined activation of Triceps Surae, Rectus Femoris, and Gluteus Maximus; TA = Tibialis Anterior; RF = Rectus Femoris; Gmax = Gluteus Maximus; and TS = Triceps Surae. Bars closer to GDI = 100 indicate more typical kinematics; GDI = 100 represents typically developing (TD) children.

In CP01-04, none of the conditions had a GDI score that surpassed the CMD threshold (Table 3, Figure 2B). However, CP05 showed a clinically meaningful improvement in the GDI score for the “PT-derived” condition, with a 7.121-point increase from the “NoStim” baseline (Table 3, Figure 2B). In CP06, all conditions exceeded the CMD threshold except for the “Gmax” condition (Table 3, Figure 2B). Figure 2A also shows that none of the conditions’ $\bar{\mathrm{GDI}}$ scores surpassed the CMD threshold.

### 3.2. CV

**Figure 3:**
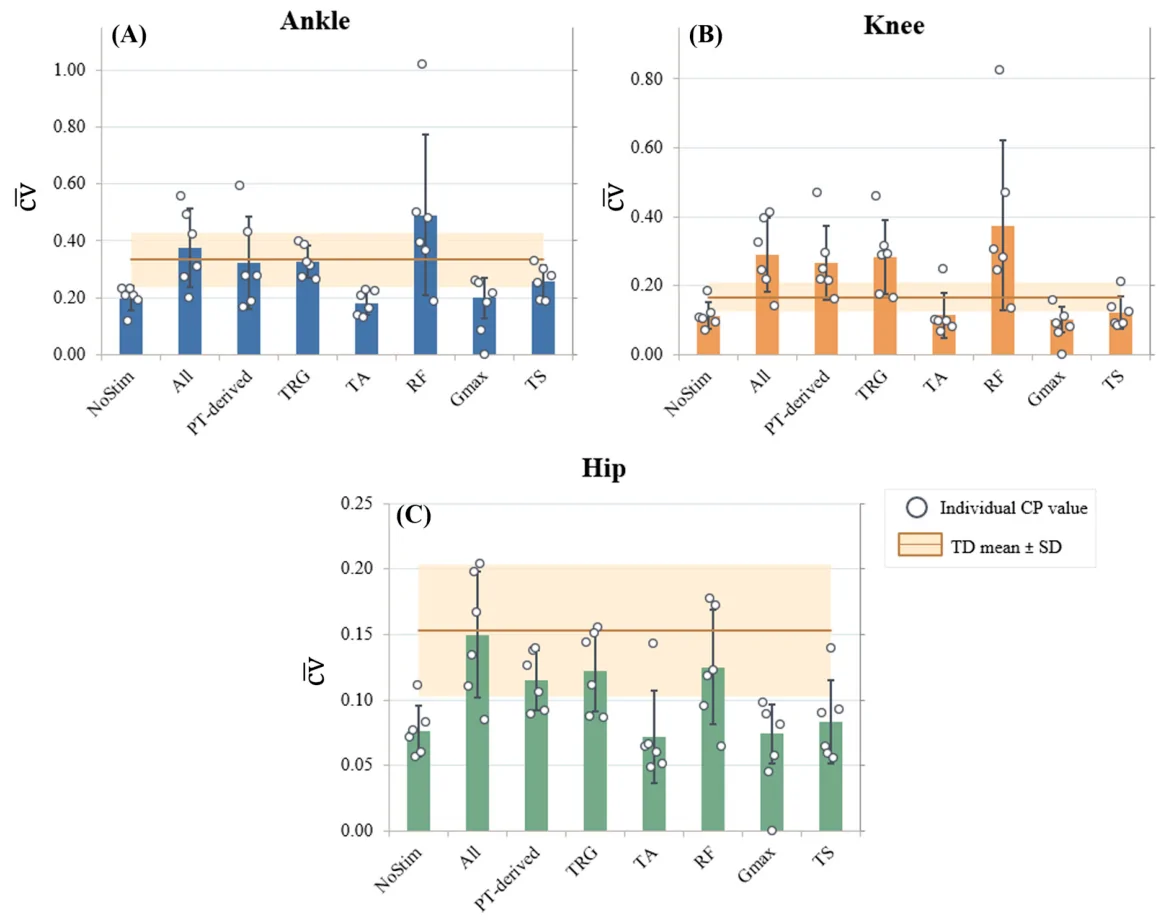
Averaged Coefficient of Variation ($\bar{\mathrm{CV}}$) for each condition across all subjects for (**A**) Ankle joint, (**B**) Knee joint, and (**C**) Hip joint. Reaching the shaded area indicates getting closer to the typically developing (TD) children’s (control group) range. Similarity is assessed based on the absolute difference between the participant and TD reference values.

As shown in Figure 3 and Table 4, the TD reference group had a higher CV values than the CP group for the hip. For the ankle (Figure 3A), $\bar{\mathrm{CV}}$ values under all three multi-muscle conditions—All, PT-derived, and TRG—fell within one standard deviation of the TD mean, whereas the NoStim value did not. The same pattern was observed for the hip (Figure 3C). For both the ankle and knee, RF produced the highest $\bar{\mathrm{CV}}$ among the stimulation conditions and exceeded the TD mean. For the knee (Figure 3B), the three multi-muscle conditions yielded similar $\bar{\mathrm{CV}}$ values.

Overall, the results indicate that “RF” stimulation produced the highest $\bar{\mathrm{CV}}$ at the ankle (closest to the TD mean) and knee, while all three multi-muscle conditions showed the closest value to the TD mean for the knee and hip joints.

### 3.3. Dynamic Time Warping per Gait Phase

#### 3.3.1. Ankle Joint

**Figure 4:**
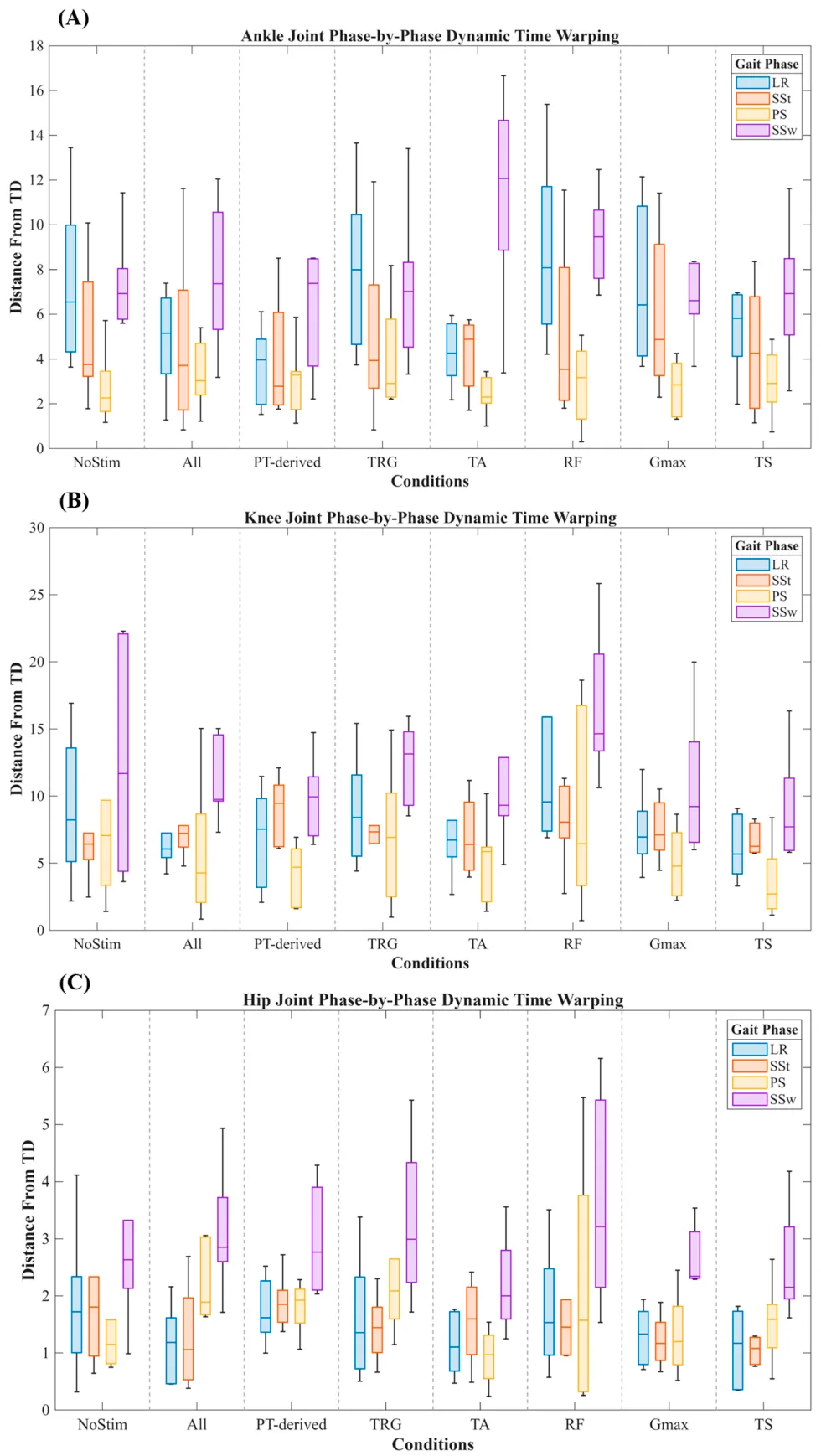
Phase-by-phase Dynamic Time Warping (DTW) distance between individuals with cerebral palsy (CP) and typically developing (TD) peers. Gait cycles were segmented into four distinct phases: Loading Response (LR), Single Support Stance (SSt), Pre-Swing (PS), and Single Support Swing (SSw). The DTW distances were calculated for the (**A**) Ankle joint, (**B**) Knee joint, and (**C**) Hip joint, compared to the TD reference group, averaged across all subjects for each experimental condition. Lower DTW values denote a smaller deviation from the TD group, indicating a higher degree of similarity in joint kinematic patterns. Although statistical analysis (α = 0.05) showed significant main effects in ankle and hip joints, pairwise post-hoc analysis did not confirm any significance between stimulation conditions and the “NoStim” baseline.

Phase-specific analysis of ankle joint DTW distances revealed a significant overall effect of stimulation condition during Loading Response (LR; Friedman χ^2^(6) = 17.64, *p* = 0.007, Kendall’s *W* = 0.49; Figure 4A). In contrast, no significant overall effects were observed during Single Support Stance (SSt; χ^2^(6) = 2.14, *p* = 0.906, *W* = 0.06), Pre-Swing (PS; χ^2^(6) = 4.64, *p* = 0.590, *W* = 0.13), or Single Support Swing (SSw; χ^2^(6) = 10.86, *p* = 0.093, *W* = 0.30).

During LR, the median DTW distance was lower for all active conditions except TRG and RF compared with NoStim. The largest reduction in median DTW was observed for the PT-derived condition (median ΔDTW = −4.57), followed by TA (ΔDTW = −1.96) and TS (ΔDTW = −1.44). The strongest pairwise effect was observed for TA (*r_rb_* = −0.90) and PT-derived (*r_rb_* = −0.71). However, none of the individual comparisons with NoStim remained statistically significant after Benjamini–Hochberg FDR correction.

#### 3.3.2. Knee Joint

No significant overall effect of stimulation condition was observed for knee joint DTW distances during any of the four gait phases (Figure 4B). Friedman tests were nonsignificant during LR (χ^2^(6) = 9.07, *p* = 0.170, Kendall’s W = 0.25), SSt (χ^2^(6) = 9.86, *p* = 0.131, *W* = 0.27), PS (χ^2^(6) = 6.93, *p* = 0.328, *W* = 0.19), and SSw (χ^2^(6) = 9.43, *p* = 0.151, *W* = 0.26).

#### 3.3.3. Hip Joint

Hip joint DTW distances demonstrated a significant overall effect of stimulation condition during Pre-Swing (PS; Friedman χ^2^(6) = 14.57, *p* = 0.024, Kendall’s *W* = 0.40; Figure 4C). No significant overall effects were observed during LR (χ^2^(6) = 8.07, *p* = 0.233, *W* = 0.22), SSt (χ^2^(6) = 6.14, *p* = 0.407, *W* = 0.17), or SSw (χ^2^(6) = 4.21, *p* = 0.648, *W* = 0.12).

During PS, the TRG condition showed the largest increase in DTW relative to NoStim (median ΔDTW = 0.79; *r_rb_* = 1.00). The All and PT-derived conditions also showed increased DTW relative to NoStim, whereas TA showed a small reduction (median ΔDTW = −0.10; *r_rb_* = −0.62). Despite the significant omnibus effect during PS, none of the individual stimulation conditions differed significantly from NoStim after FDR correction.

### 3.4. Maximum Knee Extension

**Figure 5:**
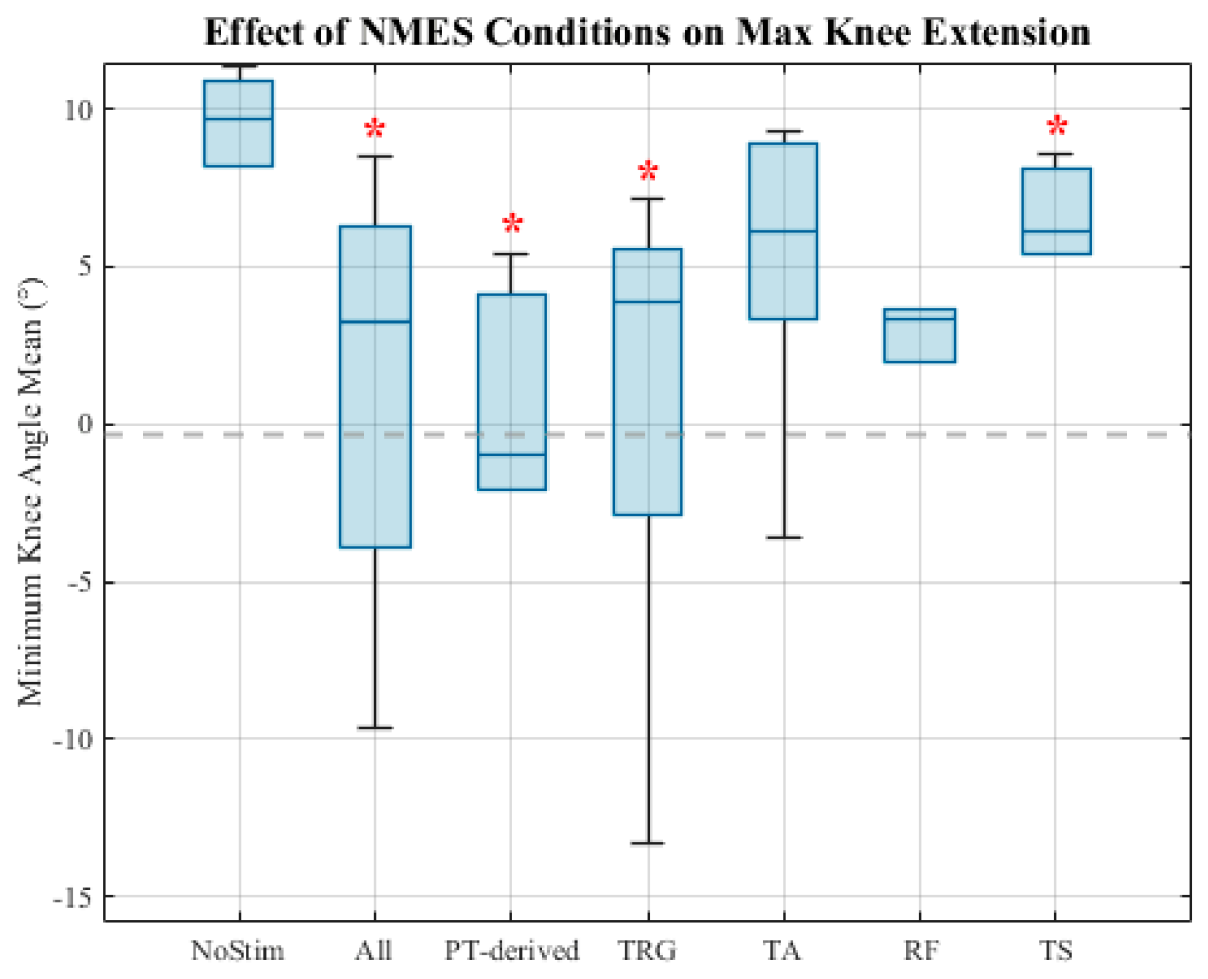
Mean values of the maximum knee extension angle (°) (corresponding to minimum knee angle) across all gait cycles and participants for each condition. The dashed gray line represents the mean maximum knee extension angle of typically developing (TD) children, serving as the control group. Positive values indicate knee flexion, while negative values indicate hyperextension. “All”, “PT-derived”, “TRG”, and “TS” conditions showed a statistically significant improvement (α = 0.05), reflected by a decrease in the flexion angle from the baseline (NoStim), and are marked with red asterisks.

The Friedman test result revealed a significant main effect of stimulation conditions on maximum knee extension (χ^2^(6) = 19.43, *p* = 0.0035) with a large main effect (Kendall’s *W* = 0.54). Post-hoc analysis with FDR correction indicated that the following conditions resulted in significantly greater knee extension (i.e., lowering the flexion angle degree) compared to the “NoStim” baseline, with all of the significant conditions showing a very large effect (Figure 5): “All” (*p* = 0.047, *r* = −0.9), “PT-derived” (*p* = 0.047, *r* = −0.9), “TRG” (*p* = 0.047, *r* = −0.9), and “TS” (*p* = 0.047, *r* = −0.9). In contrast, the “TA” (*p* = 0.219) and “RF” (*p* = 0.075) conditions were not significantly different from the baseline.

To better differentiate the effectiveness of the significant NMES conditions (i.e., All, PT-derived, TRG, and TS) and compare them with the TD group’s minimum knee angle, we calculated and compared their Median values. This measurement revealed that the lowest Median value is related to the “PT-derived” condition (*Med* = −1.004), while the medians of “All” (*Med* = 3.26), “TRG” (*Med* = 3.9), and “TS” (*Med* = 6.125) conditions were higher by at least 4.24 points. Notably, the “PT-derived” condition showed a result closer to the median minimum knee angle of the TD group, which achieved a median of −2.303.

### 3.5. Stride Length

**Figure 6:**
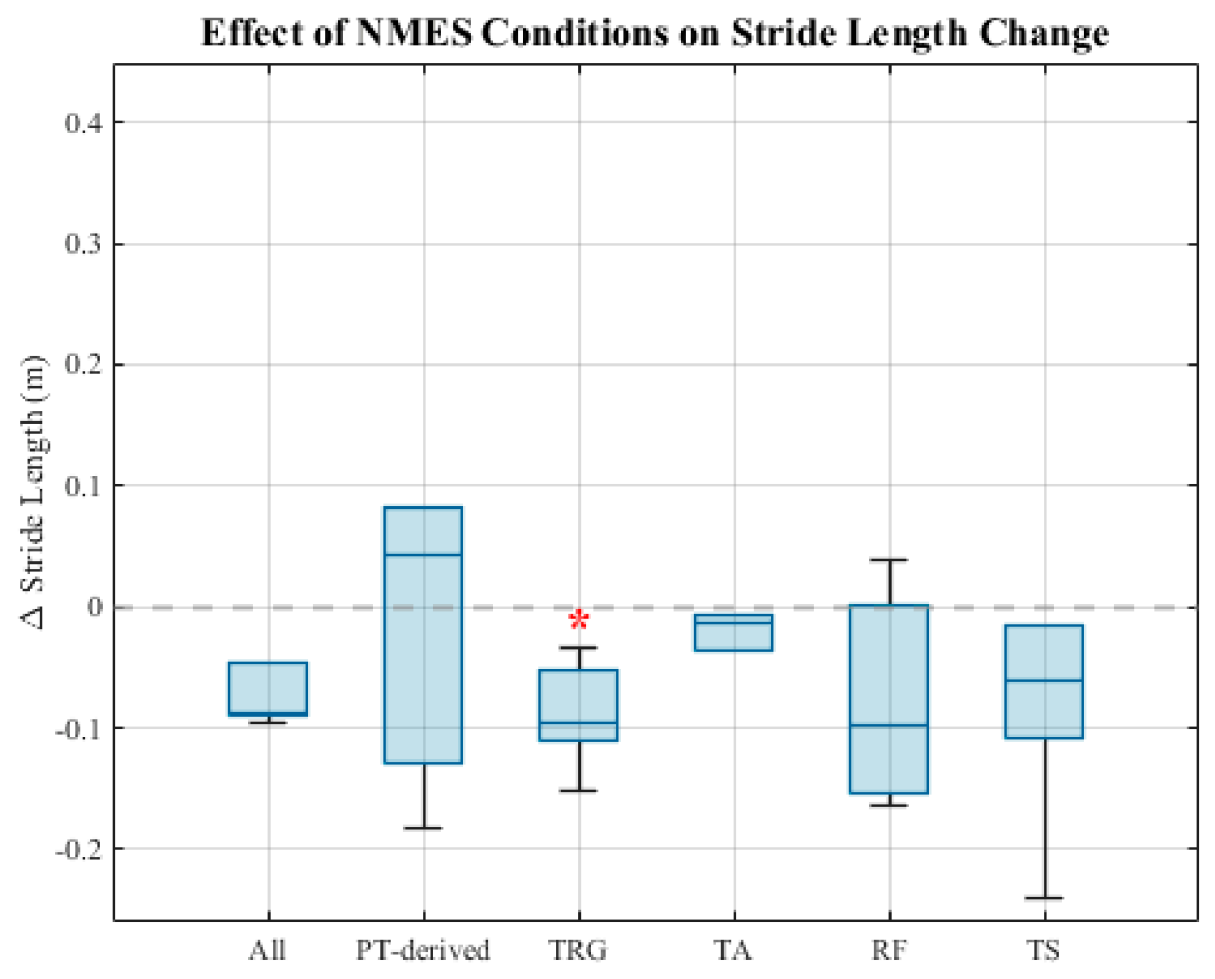
Changes in stride length (m) from the baseline (NoStim) across all gait cycles and participants for each condition. The gray dashed line represents the baseline of zero, which helps to visualize the direction of change from this baseline. Negative values indicate a decrease in stride length, while positive values represent an increase. “TRG” condition, which showed a statistically significant (α = 0.05) difference from the baseline, is highlighted with a red asterisk, although this difference was not in the direction of improvement.

Statistical analysis of the stride length data showed a significant main effect of NMES conditions (χ^2^(6)= 13.09, *p* = 0.042) with a medium main effect (Kendall’s *W* = 0.36). Post-hoc analysis indicated that this effect was driven by the “TRG” condition (*p* = 0.0312, *r* = −0.9). As shown in Figure 6, and considering the negative value of its effect size, this notable difference reflected a decrease in stride length rather than an improvement. Among all the conditions assessed, the only condition that resulted in a positive effect size was “PT-derived” (*r =* 0.13), although it was not significant (*p* = 0.84).

### 3.6. Multi-Muscle vs. Single-Muscle

**Figure 7:**
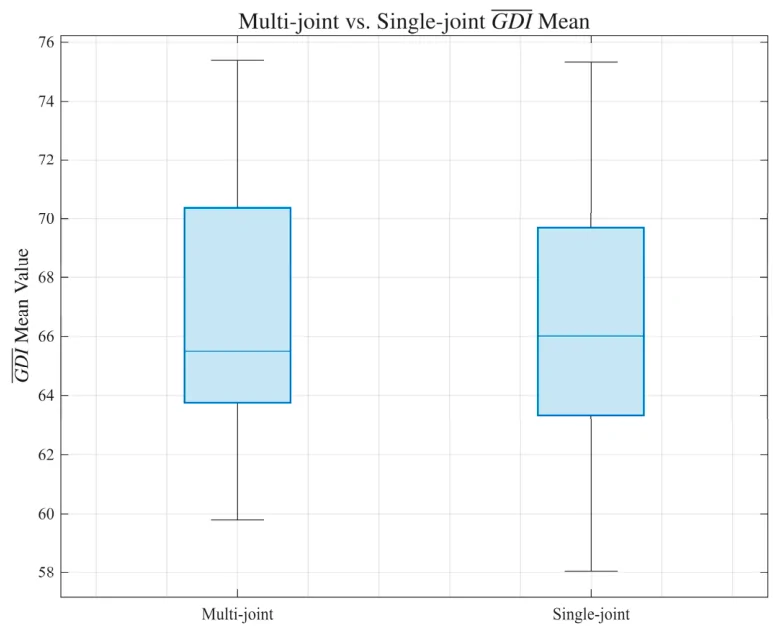
Distribution of participant-level mean Gait Deviation Index $\left(\bar{\mathrm{GDI}}\right)$ scores across multi-muscle (“All”, “PT-derived”, and “TRG”) and single-muscle (“TA”, “RF”, and “TS”) stimulation conditions for six participants. Higher $\bar{\mathrm{GDI}}$ values represent a gait pattern closer to that of typically developing (TD) peers. The two-sided exact Wilcoxon signed-rank test showed no statistically significant difference between categories (unadjusted *p* = 1.000; matched-pairs rank-biserial correlation = 0.048). The mean paired difference (multi-muscle minus single-muscle) was 0.098 (95% bootstrap CI: −0.567 to 0.860).

Two-sided exact Wilcoxon signed-rank analysis comparing participant-level mean ± SD of the $\bar{\mathrm{GDI}}$ scores between multi-muscle conditions (“All”, “PT-derived”, and “TRG”; 66.72 ± 5.54) and single-muscle conditions (“TA”, “RF”, and “TS”; 66.62 ± 5.82) did not reveal any significant difference (*p* = 1.00, *r*_rb_ = 0.048) (Figure 7). The mean paired difference was 0.098 (95% bootstrap CI: −0.567 to 0.860). Gmax was excluded from the single-muscle category average for all participants to maintain consistent protocol composition while retaining all six participants.

**Figure 8:**
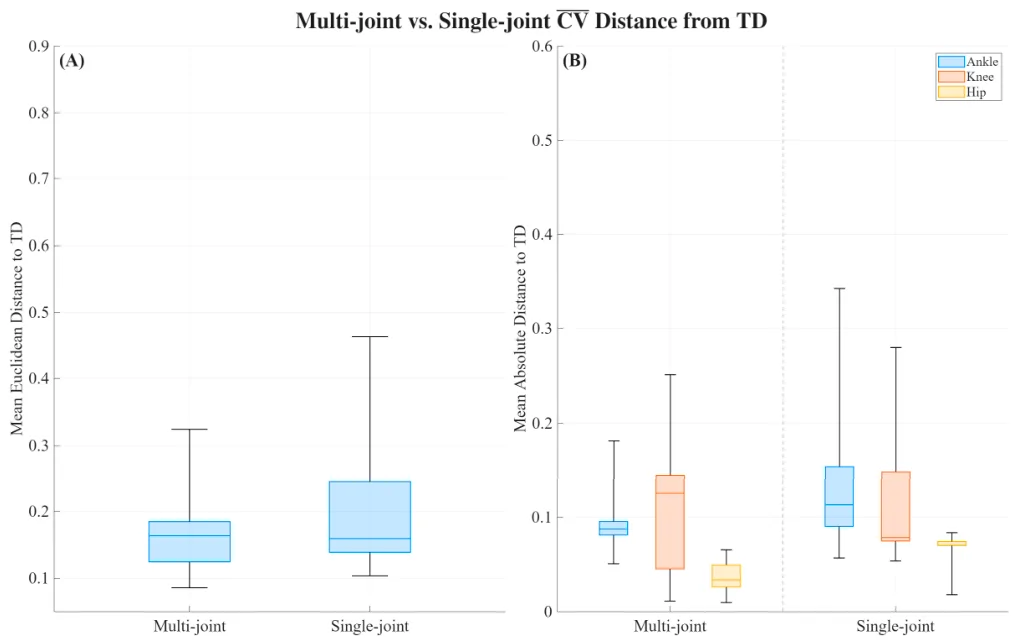
Comparison of coefficient of variation (CV) distances from typically developing (TD) reference values between multi-muscle (“All”, “PT-derived”, and “TRG”) and single-muscle (“TA”, “RF”, and “TS”) stimulation conditions for six participants. (**A**) Distribution of participant-level mean Euclidean distances from TD $\bar{\mathrm{CV}}$ values across ankle, knee, and hip joints averaged over conditions within each category. Distances were smaller under multi-muscle stimulation (unadjusted *p* = 0.0312). (**B**) Participant-level mean absolute distance of $\bar{\mathrm{CV}}$ from TD values analyzed for each joint. Distances were smaller under multi-muscle stimulation for the hip, but not statistically significant (FDR-adjusted *p* = 0.125). Lower distances indicate greater similarity to TD CV values, rather than lower movement variability. All comparisons are exploratory.

Statistical analysis of joints’ $\bar{\mathrm{CV}}$ demonstrated no significant differences between conditions after FDR correction (Figure 8B). The multi-muscle condition showed a smaller mean distance from TD at the hip, though this difference did not remain significant after Benjamini–Hochberg correction (unadjusted *p* = 0.03125, FDR-adjusted *p* = 0.125, *r*_rb_ = −1.000; mean paired difference: −0.028, 95% bootstrap CI: [−0.038, −0.017]) (Figure 8B). The ankle comparison did not reach statistical significance (unadjusted *p* = 0.5625, FDR-adjusted *p* = 0.75, *r*_rb_ = −0.333; mean paired difference: −0.048, 95% bootstrap CI: [−0.137, 0.023]), nor did the knee comparison (unadjusted *p* = 0.84375, FDR-adjusted *p* = 0.84375, *r*_rb_ = −0.143; mean paired difference: −0.002, 95% bootstrap CI: [−0.071, 0.078]). To better visualize the results, the Euclidean distance is calculated across the ankle, knee, and hip CV metrics for each subject and condition, then averaged within each category. This composite distance also did not differ significantly between groups (unadjusted *p* = 0.5625, FDR-adjusted *p* = 0.75, *r*_rb_ = −0.333; mean paired difference: −0.037, 95% bootstrap CI: [−0.151, 0.064]) (Figure 8A). All differences were calculated as multi-muscle minus single-muscle. These exploratory comparisons do not establish the general superiority of multi-muscle NMES.

## 4. Discussion

This exploratory study characterized acute biomechanical responses to seven NMES protocols in six children with CP, emphasizing within-participant changes and differences in response patterns across stimulation configurations. Three conditions targeted different combinations of lower limb muscles across multiple joints, thus creating the multi-muscle NMES protocols (All, PT-derived, TRG). The other four conditions (TA, RF, Gmax, and TS) focused on assisting only one muscle per condition, forming the single-muscle protocols.

### 4.1. Hypothesis 1: Multi-Muscle vs. Single-Muscle NMES

Our first hypothesis proposed that multi-muscle NMES would produce greater improvements in gait kinematics than single-muscle stimulation. The rationale for a multi-muscle approach is supported by the inherently multi-segmental nature of gait in children with CP. The primary purpose of this approach was to determine whether addressing the inherently multi-segmental nature of gait deviations in CP through synchronized, cross-joint NMES assistance would produce a more integrated biomechanical response and shift gait mechanics toward a pattern more closely resembling that of TD individuals.

The comparison between multi-muscle and single-muscle NMES did not identify a statistically significant difference in GDI, the primary outcome. Exploratory analyses of selected CV measures showed smaller distances from TD reference values under multi-muscle conditions at the hip joint sagittal plane angle. These observations describe differences among the protocols examined in this sample but do not establish the superiority of multi-muscle stimulation. Their interpretation should account for the small number of participants, the range of outcomes examined, and variability in individual responses.

#### 4.1.1. *CV*

Comparison of multi-muscle $\bar{\mathrm{CV}}$ and the mean Euclidean $\bar{\mathrm{CV}}$ across all the joints in multi-muscle conditions, versus that of single-muscle conditions, demonstrated no statistically significant trend for multi-muscle stimulation. When the composite distance was decomposed by joint, the multi-muscle conditions (“All,” “PT-derived,” and “TRG”) showed a smaller mean distance from TD than the single-muscle conditions at the hip, but this difference did not remain significant after Benjamini–Hochberg correction across the four CV comparisons (unadjusted *p* = 0.03125, FDR-adjusted *p* = 0.125); the ankle and knee comparisons were not significant even before correction (unadjusted *p* = 0.5625 and 0.84375, respectively).

These exploratory comparisons do not establish that multi-muscle NMES produces a more coordinated modification of movement variability across joints. However, CV does not necessarily represent a direct restoration of physiological variability, and the observed changes should therefore be interpreted as increased similarity to the TD reference rather than definitive normalization of motor control.

#### 4.1.2. *GDI*

Although the difference between the multi-muscle mean $\bar{\mathrm{GDI}}$ and that of the single-muscle did not reach statistical significance, a similar directional trend as $\bar{\mathrm{CV}}$ was observed, with multi-muscle conditions yielding higher mean scores than single-muscle conditions, though the effect size was negligible (*r_rb_* = 0.048). In detailed comparisons of clinically meaningful changes (∆GDI ≥ 5; CMD threshold), multi-muscle conditions more frequently exceeded the CMD. Specifically, in CP06, “All,” “PT-derived,” and “TRG” exceeded CMD, and in CP05, “PT-derived” exceeded CMD. In contrast, single-muscle stimulation demonstrated fewer meaningful improvements (CP06: “TS” > CMD) and was occasionally detrimental.

Despite these directional findings, the lack of a statistically significant group-level difference in GDI indicates that multi-muscle stimulation cannot be considered superior to single-muscle stimulation based on this global gait index in the present sample. These results suggest that targeted neuromuscular assistance may refine specific biomechanical deficits without immediately altering global gait pathology as quantified by GDI.

Changes in the GDI following NMES intervention in children with CP have been variable across studies and appear to depend, in part, on both participant characteristics and stimulation strategy. Bailes et al. [44] stimulated the TA muscles of 11 children with hemiplegic CP (GMFCS I–II) primarily to assist their ankle dorsiflexion. Although improvements were observed in dorsiflexion at initial contact and walking performance after a 16-week intervention, GDI did not change significantly [44]. Rose et al. [34] investigated a small cohort of children with CP using a multichannel NMES system targeting combinations of muscles, including TA, TS, RF, and Ham, and reported improvements in selected kinematic parameters; however, GDI exceeded the CMD threshold in only one of three participants [34]. In contrast, Danino et al. [23] studied children with hemiplegic CP using peroneal nerve stimulation of the TA and reported modest improvements in composite gait indices in some participants, with responses influenced by baseline gait impairment [23]. Consistent with this body of literature, our findings suggest that while stimulation, whether single-muscle dorsiflexor assistance or broader multichannel approaches, may induce joint- or phase-specific kinematic adaptations, these changes do not necessarily translate into substantial modification of overall gait pathology as quantified by GDI. This pattern underscores the possibility that targeted neuromuscular assistance may refine specific biomechanical deficits without immediately altering the multi-segment coordination reflected in global gait metrics.

#### 4.1.3. Targeted Metric

All the multi-muscle NMES conditions, including “All” and “TRG”, and the “PT-derived” and “TS” from the single-muscle protocol, produced significant improvements in maximum knee extension with large effect sizes, indicating enhanced sagittal-plane knee control during gait. Although the “PT-derived” condition resulted in slight hyperextension on average, its values remained the closest to the TD reference compared to other conditions.

Evidence regarding the effects of NMES/Functional Electrical Stimulation (FES)-assisted walking on knee extension in children with CP suggests that the most direct improvements are observed when stimulation targets the quadriceps and is timed to stance [25,27,45]. Case-based and single-participant experimental studies have reported acute increases in knee extension during MSt and reductions in crouch posture when quadriceps stimulation is synchronized with gait events [25,27]. In contrast, the majority of pediatric FES studies have focused on peroneal or TA stimulation for foot drop, where improvements are primarily observed at the ankle, and consistent changes in knee extension are not typically reported [45].

Unlike knee extension, the observed differences in stride length were significant but occurred in an unfavorable direction, resulting in shorter strides. This finding is somewhat unexpected, as improved knee extension during stance would theoretically contribute to increased limb advancement and longer stride length.

Selected outcomes showed changes in a favorable direction under multi-muscle NMES, but responses were mixed across measures and participants. These preliminary observations do not establish broader improvements in overall gait relative to single-muscle stimulation. These observations can be considered alongside previous single-joint (Knee joint) multi-muscle NMES walking studies in children with CP, including the work of Shideler et al., which explores the acute benefits of stimulating RF and vastus lateralis over stimulating RF alone [27]. Similarly, Pierce et al. applied percutaneous intramuscular FES to the TA, gastrocnemius, and their combination, finding that simultaneous stimulation of both muscles significantly improved ankle dorsiflexion during the swing phase [24]. In a larger feasibility study, Orlin et al. showed that coordinated, phase-specific stimulation of both muscles produced more consistent and statistically supported improvements than single-muscle stimulation, highlighting the potential advantage of synchronized agonist–antagonist activation over single-muscle stimulation [46].

A strictly cumulative or additive improvement across all metrics was not observed in the present acute study. This distinction likely reflects the inherently non-linear and multi-segmental nature of gait deviations in CP. Pathological gait patterns are heterogeneous and interdependent across joints [47], and improvements at one segment may alter inter-joint coordination or redistribute mechanical contributions rather than summate proportionally in global indices such as GDI or CV. Moreover, prior NMES investigations have documented considerable inter-individual variability in response to stimulation [18,19], suggesting that synchronized multi-joint activation does not guarantee uniform additive gains, particularly during short-term exposure.

Importantly, our previous longitudinal training work indicates that dosage may substantially influence functional integration [32,33]. In that study, some kinematic improvements toward TD emerged progressively over 36 sessions and were maintained at four-week follow-up, suggesting that broader additive effects may require motor adaptation over time. Although the controller updated phase-duration estimates during walking, the 75% trigger could not ensure that stimulation coincided with the intended phase onset on every stride. Stride-to-stride changes in phase duration, phase-detection error, and the delay between the stimulation command and delivered pulse could shift stimulation timing. Because the comparison of multi-muscle protocols depends on the coordination of muscle activation, these timing errors may have influenced the observed responses. The present data cannot determine the magnitude or direction of that influence [28]. The potential influence of stimulation timing and training exposure warrants further investigation. The present acute comparisons cannot determine whether optimizing these factors would produce greater global gait improvements.

### 4.2. Hypothesis 2: Individualized PT-Derived Protocol

Our second hypothesis proposed that an individualized “PT-derived” NMES protocol would outperform standardized multi-muscle conditions (All and TRG) was not statistically confirmed. Nevertheless, selected participant-specific and joint-level responses identify questions for further investigation of individualized stimulation.

First, the “PT-derived” condition was the only protocol that exceeded the CMD threshold in CP05 and was among the CMD-exceeding conditions in CP06. Furthermore, this individualized condition demonstrated: (1) the closest median knee extension angle to TD values; (2) the largest reduction in ankle DTW during LR (though non-significant after FDR correction); and (3) the only positive (though non-significant) stride length effect. These findings suggest that tailoring stimulation timing and muscle selection to subject-specific gait deviations may enhance biomechanical targeting.

However, the lack of statistically significant superiority over other multi-muscle protocols across the full cohort likely reflects: (1) small sample size (*N* = 6); (2) heterogeneity in GMFCS levels; (3) variability in muscle responsiveness to stimulation; and (4) phase-triggering limitations (75% prior-phase activation rule).

Notably, the “PT-derived” protocol’s benefits were more apparent in targeted joint-level measures than in global indices. This suggests that individualized NMES may lead to localized biomechanical corrections, which may require longer training sessions to achieve significant overall gait improvements, as seen in our case study involving two CP subjects who underwent a 12-week training program using the “PT-derived” protocol [32].

Our phase-specific DTW analysis showed a significant overall effect of stimulation conditions on ankle kinematics during LR and hip kinematics during PS. However, follow-up comparisons did not identify any individual stimulation condition that was statistically different from “NoStim” after FDR correction. During ankle LR, the PT-derived condition demonstrated the largest reduction in median DTW relative to NoStim, although this difference was not statistically significant after correction. Similarly, during hip PS, the PT-derived condition did not show a significant improvement relative to NoStim. These findings suggest that the individualized PT-derived protocol may influence specific aspects of joint-level kinematics, but the present data do not provide sufficient evidence that it consistently improves similarity to TD gait compared with the NoStim condition.

Thus, while the individualized PT-derived protocol did not statistically dominate standardized one-size-fits-all multi-muscle conditions, its directional effects on selected joint-level measures and participant-specific responses suggest that further investigation of individualized stimulation strategies is warranted.

Several limitations should be considered when interpreting these findings. First, the study included only six children with CP, aged 12–18 years, and classified at GMFCS levels II and III. This small, heterogeneous sample limits the precision and generalizability of the observed effects. Repeated measurements across stimulation conditions provide useful within-participant information but do not increase the number of independent participants. Second, both groups walked at their self-selected speed, which was chosen specifically to allow each participant to walk naturally and minimize the influence of an imposed, unfamiliar pace on gait quality and variability, rather than being fixed at a common value across participants. This approach does not eliminate the resulting difference between groups: TD speeds (0.80–1.25 m/s) were on average faster than CP speeds (0.45–0.90 m/s), overlapping only between 0.80 and 0.90 m/s. Because joint kinematics and their variability depend on walking speed, and time normalization and dynamic time warping do not remove speed-related differences in waveform shape, amplitude, or variability, similarity to the TD reference (GDI, CV distance from TD, and DTW) may reflect walking-speed differences as well as CP-related gait characteristics. Third, the numerous comparisons across global, joint-specific, and phase-specific outcomes increase the possibility of chance findings and selective emphasis on favorable results. Although multiplicity adjustments were applied to specified analyses, the overall investigation remains exploratory, and effect-size estimates may be unstable. Finally, the study assessed acute responses and cannot establish sustained functional benefit or comparative clinical efficacy. These findings should inform outcome selection and the design of future studies, with sample-size planning accounting for uncertainty in the present estimates.

Additionally, the observed reduction in stride length despite improved knee extension suggests that distal propulsion or inter-joint coordination may constrain global performance. Future work should investigate kinetic parameters, ankle push-off mechanics, and energy cost to better understand these interactions.

Finally, the muscle selection in the individualized “PT-derived” protocol was identified by our PTs based on the standardized gait videos of each participant, focusing on sagittal plane improvement. It is important that future studies incorporate more objective and reproducible frameworks, such as instrumented gait analysis metrics and surface EMG recordings, to guide stimulation prescription and improve standardization. Also, this protocol showed promising trends but did not consistently outperform standardized multi-muscle conditions. Larger trials are needed to determine whether subject-specific stimulation timing and muscle selection yield superior long-term outcomes.

Future research should focus on: (1) increasing the sample size based on power analysis; (2) longitudinal training studies to assess retention and neuroplastic effects; (3) optimization of multi-muscle stimulation timing and amplitude; (4) integration of adaptive or closed-loop control strategies; and (5) evaluation of metabolic cost and functional mobility outcomes. By advancing toward individualized, multi-channel neuromodulation strategies, future work may enhance both biomechanical correction and functional independence in children with CP.

## 5. Conclusions

This study examined the acute effects of different NMES strategies during treadmill walking in children with CP, comparing multi-muscle, single-muscle, and individualized stimulation approaches. The comparison between multi-muscle and single-muscle conditions did not identify a statistically significant difference in GDI, the primary outcome. Selected complementary measures showed differences in response direction and magnitude, with responses varying across outcomes and participants. The individualized PT-derived protocol showed favorable responses in some measures and participants, but a consistent advantage over standardized protocols was not established.

Phase-specific waveform analyses provided further insight into localized adaptations that were not always captured by global gait indices. While certain stimulation strategies influenced specific joint mechanics, these changes did not uniformly translate into improvements in overall gait performance. These findings suggest that NMES may influence specific portions of the gait cycle, but the present data do not establish a clear improvement associated with any individual stimulation strategy.

Taken together, these findings provide preliminary within-participant information about responses to different stimulation configurations. They do not establish the comparative efficacy of multi-muscle, single-muscle, or individualized NMES. Larger, prospectively designed studies with prespecified comparisons and outcomes are needed to evaluate these hypotheses and determine whether acute biomechanical responses translate into sustained functional benefits.

## Figures and Tables

**Table 1:** Detailed information on participant age, gender, height, weight, Gross Motor Function Classification System (GMFCS), self-selected walking speed (SSWS), affected side, and the assistive devices used.

Subjects	Age (yrs)	Gender	Height (m)	Weight (kg)	GMFCS	SSWS (m/s)	Affected Side	Assistive Device/Brace
CP01	15	M	1.67	32.13	III	0.60	L	Bilateral Lofstrand crutches
CP02	16	M	1.70	60.06	III	0.80	L	Bilateral Lofstrand crutches and AFO
CP03	18	M	1.70	61.97	II	0.90	R	-
CP04	12	M	1.52	41.50	II	0.75	R	-
CP05	13	F	1.31	32.13	III	0.45	L	Posterior and anterior walkers
CP06	13	F	1.44	42.53	II	0.80	L	-
TD01	16	M	1.78	71.92	-	0.80	-	-
TD02	10	M	1.46	32.55	-	0.80	-	-
TD03	10	F	1.46	31.95	-	1.20	-	-
TD04	12	F	1.59	43.25	-	1.25	-	-
TD05	12	F	1.47	36.42	-	1.00	-	-
TD06	14	F	1.55	52.61	-	1.10	-	-
TD07	13	F	1.73	56.29	-	1.10	-	-

**Table 2:** Ranges of programmed stimulation settings in the individualized PT-derived condition.

Participant	Current Range (mA)	Pulse-Duration Range (µs)
CP01	45–90	250–470
CP02	40–50	102–252
CP03	35–90	240–365
CP04	35–60	125–340
CP05	30–35	230–350
CP06	35–60	225–320

**Table 3:** Gait Deviation Index (GDI) results across all conditions and their difference from the baseline. The ∆ symbol indicates the results after subtracting NoStim. NoStim = Without any stimulation, All = Typical muscle activation patterns, PT-derived = Physical therapists’ chosen muscles, TA = Tibialis Anterior, RF = Rectus Femoris, Gmax = Gluteus Maximus, TRG = Combined activation of Triceps Surae, Rectus Femoris, and Gluteus Maximus. * Indicates exceeding the clinically relevant difference threshold (CMD ≥ 5).

Subjects	Score	NoStim	All	PT-Derived	TRG	TA	RF	Gmax	TS
**CP01**	GDI	64.317	63.874	66.429	60.979	65.125	62.952	59.398	65.89
	∆	-	−0.443	2.112	−3.338	0.807	−1.366	−4.919	1.573
**CP02**	GDI	61.879	64.883	63.747	62.723	60.575	65.520	63.242	65.03
	∆	-	3.004	1.869	0.845	−1.304	3.642	1.363	3.151
**CP03**	GDI	78.116	73.878	74.970	77.224	75.073	73.295	77.232	75.59
	∆	-	−4.238	−3.146	−0.892	−3.043	−4.821	−0.884	−2.526
**CP04**	GDI	69.966	65.265	68.077	68.310	70.636	62.122	70.209	70.79
	∆	-	−4.701	−1.889	−1.656	0.670	−7.844	0.243	0.824
**CP05**	GDI	56.942	58.979	64.062	56.379	59.133	55.999	-	59.02
	∆	-	2.037	7.121 *	−0.562	2.192	−0.942	-	2.078
**CP06**	GDI	63.989	70.054	69.740	71.392	71.114	70.075	66.325	71.27
	∆	-	6.065 *	5.750 *	7.402 *	7.124 *	6.085 *	2.335	7.281 *

**Table 4:** Results of the Coefficient of Variation (CV) in ankle, knee, and hip joints across all participants and conditions. The ∆ symbol indicates the results after subtracting from TD. NoStim = Without any stimulation; All = Typical muscle activation patterns; PT-derived = Physical therapists’ chosen muscles; TRG = Combined activation of Triceps Surae, Rectus Femoris and Gluteus Maximus; TA = Tibialis Anterior; RF = Rectus Femoris; Gmax = Gluteus Maximus; and TS=Triceps Surae. TD = typically developing children (control group).

Subjects	Joints’ CV	NoStim	All	PT-Derived	TRG	TA	RF	Gmax	TS
CP01	Ankle	0.231	0.553	0.591	0.398	0.137	0.498	0.261	0.330
	∆	-	−0.220	−0.258	−0.065	0.196	−0.165	0.072	0.003
	Knee	0.108	0.323	0.467	0.459	0.100	0.303	0.158	0.137
	∆	-	−0.157	−0.301	−0.293	0.066	−0.137	0.008	0.029
	Hip	0.072	0.110	0.126	0.144	0.064	0.095	0.098	0.090
	∆	-	0.043	0.027	0.009	0.089	0.058	0.055	0.063
CP02	Ankle	0.208	0.272	0.167	0.274	0.208	0.395	0.253	0.250
	∆	-	0.061	0.166	0.059	0.125	−0.062	0.080	0.083
	Knee	0.103	0.243	0.217	0.174	0.096	0.243	0.092	0.089
	∆	-	−0.077	−0.051	−0.008	0.070	−0.077	0.074	0.077
	Hip	0.077	0.134	0.089	0.087	0.066	0.118	0.089	0.064
	∆	-	0.019	0.064	0.066	0.087	0.035	0.064	0.089
CP03	Ankle	0.117	0.492	0.275	0.386	0.131	1.019	0.086	0.191
	∆	-	−0.159	0.058	−0.053	0.202	−0.686	0.247	0.142
	Knee	0.069	0.395	0.249	0.290	0.066	0.823	0.063	0.084
	∆	-	−0.229	−0.083	−0.124	0.100	−0.657	0.103	0.082
	Hip	0.057	0.198	0.138	0.111	0.049	0.177	0.045	0.059
	∆	-	−0.045	0.015	0.042	0.104	−0.024	0.108	0.094
CP04	Ankle	0.208	0.421	0.188	0.313	0.160	0.478	0.181	0.188
	∆	-	−0.088	0.145	0.020	0.173	−0.145	0.152	0.145
	Knee	0.120	0.412	0.215	0.293	0.098	0.469	0.111	0.091
	∆	-	−0.246	−0.049	−0.127	0.068	−0.303	0.055	0.075
	Hip	0.059	0.204	0.106	0.155	0.060	0.172	0.057	0.055
	∆	-	−0.051	0.047	−0.002	0.093	−0.019	0.096	0.098
CP05	Ankle	0.229	0.199	0.432	0.323	0.228	0.366	-	0.301
	∆	-	0.134	−0.099	0.010	0.105	−0.033	-	0.032
	Knee	0.183	0.218	0.295	0.315	0.247	0.280	-	0.209
	∆	-	−0.052	−0.129	−0.149	−0.081	−0.114	-	−0.043
	Hip	0.111	0.167	0.140	0.151	0.143	0.123	-	0.140
	∆	-	−0.014	0.013	0.002	0.010	0.030	-	0.013
CP06	Ankle	0.191	0.306	0.275	0.265	0.221	0.187	0.215	0.277
	∆	-	0.027	0.058	0.068	0.112	0.146	0.118	0.056
	Knee	0.095	0.139	0.160	0.165	0.080	0.132	0.081	0.125
	∆	-	0.027	0.006	0.001	0.086	0.034	0.085	0.041
	Hip	0.083	0.084	0.092	0.086	0.051	0.065	0.081	0.093
	∆	-	0.069	0.061	0.067	0.102	0.088	0.072	0.060
TD	Ankle	0.333 ± 0.096							
	Knee	0.166 ± 0.042							
	Hip	0.153 ± 0.050							

## Data Availability

The data presented in this study are available upon request from the corresponding author due to privacy and ethical restrictions involving minor participants.

## Supplementary Materials

The following supporting information can be downloaded at: https://www.mdpi.com/article/10.3390/bioengineering13101193/s1: Figure S1: Stimulation program for each participant. A heat map shows the pulse duration (μs) used for each gait phase (column) and muscle group (row) and current (mA) used for each muscle group. For each muscle group, there is a left side (top row) and right side (bottom row). Darker colors are associated with higher pulse duration in red and higher current in blue. Black indicates no stimula tion. 1: loading response; 2: mid-stance; 3: terminal stance; 4: pre-swing; 5: initial swing; 6: mid swing; 7: terminal swing; mA: current; P: plantarflexors; D: dorsiflexors; Q: quadriceps; H: ham strings; G: gluteals [30]; Table S1: Participant-specific Physical Therapist-derived (PT-derived) NMES condition treatment targets, muscle phase selections, prescription rationale, and relevant modifications.

## Abbreviations

CP	Cerebral Palsy
NMES	Neuromuscular Electrical Stimulation
TD	Typically Developing
PT	Physical Therapist
GDI	Gait Deviation Index
CV	Coefficient of Variation
DTW	Dynamic Time Warping
CMD	Clinically Meaningful Difference
SD	Standard Deviation
GMFCS	Gross Motor Function Classification System
IRB	Institutional Review Board
SSWS	Self-Selected Walking Speed
10MWT	10-Meter Walk Test
IMU	Inertial Measurement Unit
GPD	Gait Phase Detection
LR	Loading Response
MSt	Mid-Stance
TSt	Terminal Stance
PS	Pre-Swing
ISw	Initial Swing
MSw	Mid-Swing
TSw	Terminal Swing
SSt	Single Support Stance
SSw	Single Support Swing
HS	Heel-Strike
TO	Toe-Off
vGRF	Vertical Ground Reaction Force
MT	Motor Threshold
EMG	Electromyography
FES	Functional Electrical Stimulation
TA	Tibialis Anterior
RF	Rectus Femoris
Gmax	Gluteus Maximus
Ham	Hamstring
TS	Triceps Surae
TRG	Combined activation of Triceps Surae, Rectus Femoris, and Gluteus Maximus
FDR	False Discovery Rate
W	Kendall’s Coefficient of Concordance
*r*_rb_	Matched-Pairs Rank-Biserial Correlation
