# Supplementary material for: Exploring the Acute Effects of Stimulating Different Muscle Combinations During Neuromuscular Electrical Stimulation-Assisted Treadmill Walking in Children with Cerebral Palsy
Source: Bioengineering (Basel). 2026 Oct 14;13(10):1193. doi: 10.3390/bioengineering13101193 (PMC13701523; doi:10.3390/bioengineering13101193)
Supplement: Supplementary file 1 [file bioengineering-13-01193-s001.zip › bioengineering-4574575-supplementary.pdf]

**Supplementary Materials:**

**Table S1.** Participant-specific Physical Therapist-derived (PT-derived) NMES condition treatment targets, muscle-phase selections, prescription rationale, and relevant modifications.

| Participant | Principal gait deviations or treatment targets | Final muscles and stimulation phases | Clinical rationale and modifications |
|---|---|---|---|
| **CP01** | Reduced push-off; insufficient toe clearance during swing; insufficient hip and knee extension during stance; restricted forward progression of the limb | Plantarflexors: PSw; dorsiflexors: ISw–TSw; quadriceps: TSw; gluteals: MSt–TSt | Plantarflexor stimulation was restricted to PSw because the original MSt–PSw timing limited forward limb progression. Hamstring stimulation was removed. Quadriceps stimulation was moved from LR–MSt to TSw to assist limb progression and terminal-swing positioning. Gluteal stimulation targeted hip extension during stance. |
| **CP02** | Reduced push-off; insufficient toe clearance; inadequate hip external rotation; insufficient knee extension during stance; insufficient knee flexion and forward limb progression near the end of stance | Plantarflexors: MSt–PSw; dorsiflexors: ISw–TSw; quadriceps: LR–MSt and PSw; hamstrings: TSt; gluteals: TSw–TSt across the gait-cycle transition | Plantarflexors targeted push-off; dorsiflexors targeted toe clearance; quadriceps targeted knee extension and assisted progression from PSw; hamstrings targeted knee flexion near the end of stance; gluteals targeted hip external rotation and stability. The secondary quadriceps strategy during PSw was tested and retained. No further modification was required. |
| **CP03** | Reduced push-off; insufficient toe clearance; insufficient hip and knee extension during stance; reduced stance stability; insufficient limb extension near the end of swing | Plantarflexors: PSw; dorsiflexors: ISw–TSw; quadriceps: TSw; gluteals: MSt–TSt | The original quadriceps stimulation during stance caused the legs to lock in extension; therefore, quadriceps stimulation was retained only during TSw. Plantarflexor stimulation was also restricted to PSw. A proposed hamstring strategy during swing was not used. |
| **CP04** | Reduced push-off; insufficient toe clearance; inadequate hip external rotation during stance; insufficient knee extension during early stance, particularly associated with difficulty unlocking the left knee | Plantarflexors: MSt at reduced intensity and TSt–PSw at functional intensity; dorsiflexors: ISw–TSw; quadriceps: LR at reduced intensity and MSt at functional intensity; gluteals: MSt–TSt | Plantarflexors targeted controlled shank progression followed by push-off; dorsiflexors targeted toe clearance; gluteals targeted hip external rotation; quadriceps targeted knee extension after verbal or physical cueing to unlock the knee. The proposed quadriceps strategy during TSw was not used. |
| **CP05** | Reduced push-off; frequent toe dragging and insufficient toe clearance; insufficient hip and knee extension during stance; reduced stability during weight bearing | Plantarflexors: MSt–PSw; dorsiflexors: ISw–TSw; quadriceps: LR–MSt; gluteals: excluded; hamstrings: not used | Plantarflexors targeted push-off, dorsiflexors targeted toe clearance, and quadriceps targeted knee extension during early stance. Gluteal stimulation was removed because the participant could not tolerate it during walking, even after application of EMLA cream. The proposed secondary strategies were not used. |
| **CP06** | Reduced push-off; toe dragging and midfoot or forefoot initial contact; insufficient hip extension | Plantarflexors: TSt–PSw; dorsiflexors: PSw–TSw and LR according to Appendix Table B.6; | Plantarflexors targeted push-off; dorsiflexors targeted toe clearance and heel contact; quadriceps targeted terminal-swing knee extension and |

| | | | |
|---|---|---|---|
| | during stance; insufficient knee extension near the end of swing and inadequate limb positioning for weight acceptance | quadriceps: MSw–TSw and LR according to Appendix Table B.6; gluteals: LR–TSt | preparation for weight acceptance; gluteals targeted hip extension during stance. The dissertation narrative states that quadriceps pulse duration was increased to 300 µs, whereas Appendix Table B.6 reports 250 µs. This discrepancy must be resolved from the original experimental record. |

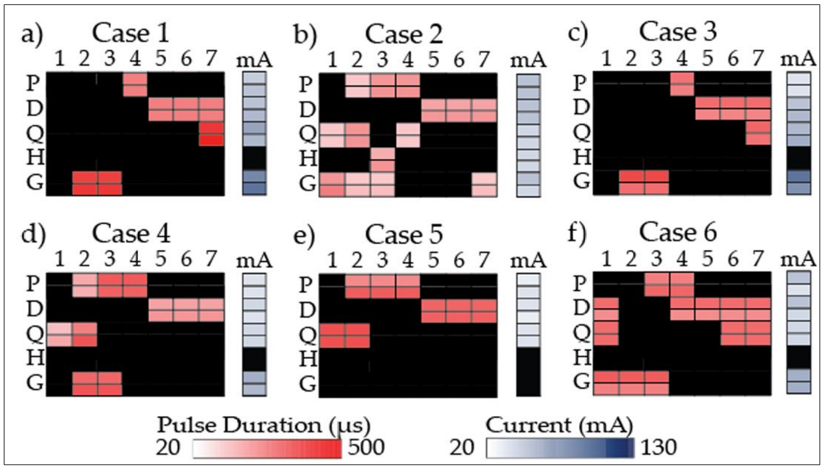


**Figure S1.** Stimulation program for each participant. A heat map shows the pulse duration (µs) used for each gait phase (column) and muscle group (row) and current (mA) used for each muscle group. For each muscle group, there is a left side (top row) and right side (bottom row). Darker colors are associated with higher pulse duration in red and higher current in blue. Black indicates no stimula tion. 1: loading response; 2: mid-stance; 3: terminal stance; 4: pre-swing; 5: initial swing; 6: mid swing; 7: terminal swing; mA: current; P: plantarflexors; D: dorsiflexors; Q: quadriceps; H: ham strings; G: gluteals [1].

**Reference:**

[1] Zahradka N, Behboodi A, Sansare A, Lee SCK. Evaluation of Individualized Functional Electrical Stimula-tion-Induced Acute Changes during Walking: A Case Series in Children with Cerebral Palsy. Sensors. 2021 Jun 29;21(13):4452. doi:10.3390/s21134452.
